# Cost and Distribution of Hysterectomy and Uterine Artery Embolization in the United States: Regional/Rural/Urban Disparities

**DOI:** 10.3390/medsci5020010

**Published:** 2017-05-16

**Authors:** Marquisette Glass Lewis, Olúgbémiga T. Ekúndayò

**Affiliations:** 1 Belhaven University-Atlanta, 4151 Ashford Dunwoody Rd, #130, Atlanta, GA 30319, USA; 2 MPH Program, Department of Public Health and Health Administration, College of Health Science and Public Health, Eastern Washington University, 668 North Riverpoint Blvd., Room 230, Spokane, WA 99202, USA

**Keywords:** uterine fibroids, reproductive health, care utilization, hysterectomy, uterine artery embolization, healthcare, disparities, gynecological conditions, women’s health

## Abstract

Hysterectomy, the driving force for symptomatic uterine fibroids since 1895, has decreased over the years, but it is still the number one choice for many women. Since 1995, uterine artery embolization (UAE) has been proven by many researchers to be an effective treatment for uterine fibroids while allowing women to keep their uteri. The preponderance of data collection and research has focused on care quality in terms of efficiency and effectiveness, with little on location and viability related to care utilization, accessibility and physical availability. The purpose of this study was to determine and compare the cost of UAE and classical abdominal hysterectomy with regard to race/ethnicity, region, and location. Data from National Hospital Discharge for 2004 through 2008 were accessed and analyzed for uterine artery embolization and hysterectomy. Frequency analyses were performed to determine distribution of variables by race/ethnicity, location, region, insurance coverage, cost and procedure. Based on frequency distributions of cost and length of stay, outliers were trimmed and categorized. Crosstabs were used to determine cost distributions by region, place/location, procedure, race, and primary payer. For abdominal hysterectomy, 9.8% of the sample were performed in rural locations accross the country. However, for UAE, only seven procedures were performed nationally in the same period. Therefore, all inferential analyses and associations for UAE were assumed for urban locations only. The pattern differed from region to region, regarding the volume of care (numbers of cases by location) and care cost. Comparing hysterectomy and UAE, the patterns indicate generally higher costs for UAE with a mean cost difference of $4223.52. Of the hysterectomies performed for fibroids on Black women in the rural setting, 92.08% were in the south. Overall, data analyzed in this examination indicated a significant disparity between rural and urban residence in both data collection and number of procedures conducted. Further research should determine the background to cost and care location differentials between races and between rural and urban settings. Further, factors driving racial differences in the proportions of hysterectomies in the rural south should be identified to eliminate disparities. Data are needed on the prevalence of uterine fibroids in rural settings.

## 1. Introduction

### 1.1. Distribution of Uterine Fibroids

Studies indicate that 30% to 50% of women 25 years to 49 years old, and 50% of menopausal women in the U.S.A were susceptible to developing uterine fibroids that needed medical attention [1,2,3]. Cain-Hielsen et al [4] also reported a cumulative incidence of uterine fibroids of 70% among white women and over 80% among African American women. Women who have severe uterine fibroids may experience a change in lifestyle due to heavy bleeding, pelvic pain, and bulk pressure causing an enlarged abdomen [5,6]. After a diligent search of the literature, the epidemiological distribution of the prevalence of uterine fibroids in the U.S. has not been reported with regard to its distribution by rural/urban patterns. It is also apparent that literature indicating the prevalence of uterine fibroids has been mostly based on research data from 1997 through to 2002.

### 1.2. Management Options

#### 1.2.1. Hysterectomy

Hysterectomy remains the most frequent surgical procedure to treat uterine fibroids among women who develop severe symptoms during their childbearing years [2,7,8,9,10,11]. A study of patient data for the period of 1998 to 2010 indicated that 7,438,452 inpatient hysterectomies were performed in the United States [12,13], 40% of which were to treat patients for uterine fibroids. In 2001 through 2003, women who had abdominal hysterectomy had a longer length of stay [14]. Hysterectomies performed to treat uterine fibroids accounted for $1.5 billion in annual hospital costs [15].

However, there is considerable anxiety among women about losing their uteri. Thus, many would prefer alternative procedures to remove the fibroid, saving the uterus, relieving them of the pain, and providing a sense of relief [6,12,16,17,18,19]. In addition, better quality of life outcomes have been correlated with these alternative procedures [6,20,21,22,23,24].

#### 1.2.2. Alternative Options

There is ongoing debate regarding whether women with severe uterine fibroids should have a hysterectomy procedure instead of alternatives, knowing that hysterectomy involves permanent removal of a key reproductive organ [20,25,26,27,28]. Studies [27,29,6,24] indicate other alternative management approaches, including prescription drugs, uterine artery embolization (UAE), Magnetic Resonance Guided Focused Ultrasound (MRgFUS), and myomectomy. Goodwin et al. asserted that approximately 25,000 UAE procedures were being performed worldwide annually by 2008 [30]. Memtsa and Homer suggested that UAE is a safe option for preserving the uterus and improving symptoms [17]. Many factors go into the decision to have a UAE, including age, pregnancy, and being reproductive [31,32,12]. After one year of having a UAE procedure, patients had shorter length of stay, with 15% to 32% of women having a repeat embolization, or selecting to have a hysterectomy within two to five years [30,33,34,35]. However, most studies agree that the five-year re-intervention rate of over 30% was very high, compared to surgery [36,27,26,23].

### 1.3. Costs

According to Cardozo et al., uterine fibroids were estimated to cost $5.9 billion to $34.4 billion yearly, with complications contributing significantly to the economic burden [37]. Soliman et al. estimated that the direct and indirect annual cost of uterine fibroid was $11,717–$25,023 per patient per year [38].

However, the research literature is not definitive regarding cost level advantage or disadvantage between hysterectomy and UAE. Goldberg et al. (2007) found the mean hospital cost difference from 2000 to 2002 for abdominal hysterectomy and UAE was between $2707 and $5707 higher for hysterectomy [39]. According to Volkers et al. (2008), from 2002 to 2004 the mean total cost for hysterectomy and UAE was $18,563 and $11,626, respectively [40]. Direct in-hospital cost and estimated mean difference for absence from work for hysterectomy were $8313 and $7718, respectively. For UAE, the same items cost $6688 and $3107, respectively. However, Carls et al. found that the average cost from 1999 to 2004 was $15,180 for abdominal hysterectomy and $16,430 for UAE [41]. Cain-Nielsen et al. found that the mean cost of UAE was $18,653, while the five-year quality of life survival was 3.943 [4]. Kong et al. found life time costs were different ($22,968 and $11,253), while quality of life years (22.75 and 22.54) were not significantly different between UAE and hysterectomy, respectively [16]. According to Chen et al. (2016), UAE was more cost-effective than MRgFU, hysterectomy, and myomectomy at commonly accepted willingness-topay thresholds [42]. However, Borah et al. [43] and Gupta et al. [34] independently determined that there were no significant differences between patients who had UAE or other procedures.

#### 1.3.1. Care Systems Efficiency Cost Factors

Patients that experience symptoms after hysterectomy or UAE accumulate hospital costs [44,4,38]. Women who choose to have an inpatient invasive procedure will spend more time from work due to complications [45,46].

Direct costs are associated with hospital stay, magnetic resonance imaging (MRI), blood work, and prescriptions during and after invasive and non-invasive procedures [47]. Indirect cost is mainly the time spent away from work while recovering from having the procedure. According to Lee et al., 2014 [48], patients that have uterine fibroids will incur increased costs, both direct and indirect. The REST trial [44] concluded that patients must balance between reduced length of stay for UAE and the risk for increased complications or treatment failure when considering overall cost.

#### 1.3.2. Cost Finance

According to Hartmann et al. [45] and Lee et al. [48], women who have Medicare or Medicaid are eligible for hysterectomy and UAE. Private insurance carriers accrue large amounts of indirect cost when approving either UAE or hysterectomy [44,49]. According to the literature, these are mainly driven by efficiency costs, such as patient’s type of insurance [41,49], repeated procedures, symptoms reoccurrence especially with alternative approaches, complications [43,50], and longer in-patient hospital stays. Based on their findings, Hakim et al. [51], and Taran et al. [52] stated that hospital care and type of insurance played a significant role in treatment cost. 

#### 1.3.3. Why Cost is Important

Women may need assistance in paying for alternative treatment methods, whether or not they are candidates for the procedures, if the procedure is cost effective and/or there are reduced procedural complications with improved quality of life. Research indicates employment, health insurance and financial status are determinants of women’s financial ability to access alternative options [49,53]. Also, economic analyses indicate that aggregate cost savings are possible, with less invasive procedures predicting potential cost savings [25,54]. However, other considerations for cost traditionally attributed to economic activities that can provide a basis for understanding the dynamic mechanisms for cost differences, forecasting cost modulations, and making service decisions, have not been robustly explored in the health literature. Such considerations include service location, economic affordability related to personal income, primary payer characteristics such as out of pocket payment, third party payment, customer demographic and economic characteristics, and demand related to service volume.

## 2. Study Purpose

The preponderance of research on the epidemiological distribution of uterine fibroids, and surveillance of the condition and its management, has been based on data that are either outdated, do not examine distribution by rural/urban setting, or are centered on few sources. This does not provide robust information for planning to eliminate disparities. Although most cost factors in the literature indicate significant differences, they are mainly structural and relate to care effectiveness or efficiency. However, it is unclear whether there are cost differences by other features, including geographical location, race, insurance type and income among women who have these procedures. Such information can be tailored more closely to potential cost reduction or cost control interventions, as well as planning. The purpose of this study was to determine, describe and compare the cost of UAE and classical abdominal hysterectomy related to race, income level, primary payer, region, and location.

## 3. Materials and Methods

### 3.1. Study Sample

The study population consisted of 35 to 50 year-old patients who underwent hysterectomy (HYS) or uterine artery embolization (UAE) from 2004 to 2008 for benign uterine fibroids in the west, south, east, and northeast hospital regions of the United States, in both urban and rural settings. Data were obtained from the Agency for Health Care Research and Quality’s (AHRQ) Healthcare Cost and Utilization Project (HCUP), an inpatient database of hospital stays. Within the HCUP, which uses International Classification of Diseases-9 (ICD-9 CM) codes, the National Inpatient Sample (NIS; formerly Nationwide Inpatient Sample) stratifies 20% of hospital discharges (http://www.hcup-us.ahrq.gov/nisoverview.jsp#about) and includes data on 8 million patients from 1056 U.S. hospitals and emergency room visits.

### 3.2. Variables and Trimming

Data were extracted for clinical history, clinical findings, demographic characteristics, age at the time of procedure, type of procedure, adverse complications, costs of care, length of stay, insurance, source of referral, hospital region, and hospital location (Table 1 and Table 2).

#### 3.2.1. Cost

To detect significant differences in distribution, the modal costs were categorized (as a new variable) to less than $5000; $5000 to $50,000; and greater than $50,001. Frequency distribution for these categories showed that valid cases less than $5000 were 1.7%, $5000 to $50,000 were 96.4% (134,323), and above $50,000 was 1.9%. The sample in the range of $5000 to $50,000 was therefore selected for analyses and the rest were trimmed as outliers, including 3731 missing cases.

#### 3.2.2. Race

The data set of 143,078 cases showed White: 38.9%, African American: 20.9%, and Hispanic: 9.7%, while proportions of Asian/Pacific Islanders (2.3%) and Native American women (0.2%) were negligible. Thus, race was recoded into four categories, with White (38.9%), African-American/Black (20.9%), Hispanic (9.7%), and Asian/PI, Native American, Other, and Unknown, grouped into one category of “Other”, representing 30.5% of the sample.

#### 3.2.3. Household Income

The median household income categories were $1–$38,999 (24.6%), $39,000–$44,999 (24.2%), $48,000–$62,999 (25.2%), and $63,000 (26.0%). All income categories were analyzed after trimming.

#### 3.2.4. Hospital Location

Hospital location, distributed by rural and urban, indicated a total of seven untrimmed cases of UAE nationwide in the rural setting. Thus, rural UAE was eliminated from analyses due to very low sample size. Thus, inferences assume only urban settings.

#### 3.2.5. Primary Payer

Insurance was coded as PAY1, Primary Payer and measured as 1 = Medicare, 2 = Medicaid, 3 = Private including (Health Maintenance Organization) HMO, 4 = Self-Pay, 5 = No Charge, and 6 = other.

### 3.3. Diagnostic Codes

Clinical findings for ICD-9 CM diagnostic codes based on discussions with AHRQ for this study were Sub-mucous Leiomyoma (218.0), Intramural Leiomyoma (218.1), Sub-serous Leiomyoma (218.2), Uterine Leiomyoma Not Otherwise Specified (NOS, 218.9), and part unspecified (219.9). Hysterectomy principal procedures codes used were: Laparoscopic Supra Cervical Hysterectomy (68.31), other subtotal abdominal hysterectomy (68.39), Laparoscopic Total Abdominal Hysterectomy (68.41), Total Abdominal Hysterectomy NEC/NOS (68.49), Laparoscopic Assisted Vaginal Hysterectomy (68.51), and Other Vaginal Hysterectomy (68.59). The variable Inject/Infuse Not Elsewhere Classified (NEC, 99.29) was used for Uterine Artery Embolization/Uterine Fibroid Embolization (Table 2).

### 3.4. Data Analysis

Frequency analyses were performed to determine distribution of variables by race, location, region, length of stay, primry payer, complications, cost and procedure. Based on frequency distributions, race, income, length of stay, insurance coverage, and cost were trimmed and categorized as described. Crosstabs were used to determine cost distributions by region, place/location, for procedure, race, and primary payer. For abdominal hysterectomy, 9.8% of the sample were performed in rural locations accross the country. Therefore, all inferential analyses and associations for UAE were assumed for urban locations only.

*T*-tests were performed to determine the difference in mean costs between rural and urban setings for both UAE and hysterectomy.

## 4. Results

### 4.1. Care Distribution

After trimmings, procedures across the U.S. were distributed by region (northeast, midwest, south and west), and location (rural or urban).

Overall, 118,082 hysterectomies (Table 2) were performed on fibroids in the urban setting (9.257 times that of rural hysterectomies), the largest number being in the urban south at 48,870 (more than twice any other region), while 12,756 were performed in the rural setting (47.8% in the south region), with an overall mean cost of $17,555.39 in urban settings, and $13,838.59 in the rural.

A total of 1943 UAE procedures were performed in the urban setting, with only 5 (7 before trimming) in the rural areas (3 northeast and 2 south); thus, rural UAEs were excluded from further analyses due to very low numbers (Table 1). The overall mean cost of UAE was $20,992.56 in the urban area and $23,452.70 in the rural area.

#### 4.1.1. Race

By race and ethnicity, there were overall 45,908 urban (37.3% in the south) and 5997 rural (33.5% in the south) hysterectomies for white women. For Black patients, 24,718 hysterectomies (58.84% of white urban hysterectomies) were performed in the urban setting (60.5% or 14,949 in the south) and 1844 in the rural setting (92.08% or 1698 in the south). For Hispanics, there were 11,829 urban hysterectomies (43.49% or 5145 in the south). Native Americans, Hawaiian and Pacific Islanders, Asians and those of mixed/undetermined race formed the “Other” category because their individual numbers were small compared to the main groups. This group of “Other” constituted respectively 36,431 and 4529 of urban and rural hysterectomies. Among the “Other” urban group, 38.95% (14,193) were in the midwest region, while in the rural setting, 49.13% (2239) were from the south region.

#### 4.1.2. Household Income

Overall, total number of fibroid cases undergoing either UAE or Hysterectomy (HYS) was 143,078 (1.6% or 2352 UAE) with total cost for the entire hospital stay ranging from $0.00 to $1,254,361.00. The costs at this point did not show any significant mode or modes. After categorization, 96.4% of the patients (134,323), representing the income range of $5000 to $50,000, were selected. By income, of the 130,838 hysterectomies, 90.36% (115,828) were performed in the urban setting, with the highest number (47,843 or 41.29%) in the southern region (see Table 1 and Table 2). Of those performed in the southern region, the highest number (13,645, or 27.92%) were performed on those whose annual household income was less than $38,999, while 25,832 (21.88%) of all urban hysterectomies were in the same income category. Of the 12,756 who had hysterectomies in the rural setting however, 48% (6168) were in households that earned less than $38,999 annually, with 66% (4120) of those being performed in the southern region. In the rural setting, 49.92% of those who had hysterectomy had an annual household income of less than $38,999. Overall, 10,628 (86.01%) of those who had hysterectomy in the rural setting earned an annual household income of less than $47,999. In the urban setting however, the highest proportion of the 115,858 who had hysterectomy (27.86%, or 32,272) had an annual household income greater than $63,000, while 54.42% had $48,000 or more. The same pattern is indicated in all the regions except for the south, where, in both urban and rural settings, women in low income households tended to have hysterectomies.

### 4.2. Cost of Care

The distribution and mean cost of care by region and location (Table 3) indicated a mixed pattern, with the overall mean cost for both UAE and hysterectomy in the urban setting ($17,611.03, *n* = 120,025) being significantly higher (t = 53.433, *p* = 0.000) than that of the rural ($13,387.54, *n* = 12,761).

The cost of UAE in the northeast region indicated much higher levels in the rural setting (notwithstanding the large differences in frequencies) than the urban ($30,307.88 vs. $19,971.66; t = −1.963, *p =* 0.050), while in the south region, the opposite was the case ($13,169.94 vs. $20,985.01; t = 9.528, *p* = 0.002).

For hysterectomy, the costs indicated a pattern of higher costs in the urban setting, even though the volume of cases was much higher in that setting, the highest difference being in the northeast region ($6074.14) and the lowest in the south ($3039.47). Overall, the difference in cost between the rural and urban setting was different between UAE and HYS, with mean cost difference differing widely between the northeast and south regions (−$10,336.22 vs. $7815.07; overall: $2460.14). Comparing hysterectomy and UAE, the patterns indicate generally higher costs for UAE with a mean cost difference of $4223.52.

### 4.3. Primary Payer

After trimmings, for UAE, 1943 patients were urban and 5 rural. All of the rural cases were private/HMO or self-pay. Of the urban, 87.0% (1691) were private, HMO or self-pay; while Medicare, Medicaid or no charge, and “Other” category were 10.7% (208) and 2.3% (44), respectively.

For abdominal hysterectomy, 118,076 (89.5%) patients were urban while 12,756 were rural. Of the rural, 78.8% (10,050) were private/HMO or self-pay, 15.8% (2019) were Medicare, Medicaid or no charge, and 5.4% (687) were “Other”. Of the urban, 85.0% (100,381) were private HMO or self-pay, while Medicare, Medicaid or no charge, and “Other” category were 10.8% (12,764) and 4.2% (4931), respectively.

## 5. Discussion

### 5.1. Cost of Care

Significantly, cost of care was generally lower in the rural than urban setting, notwithstanding the 9:1 ratio of the volume of cases performed in the urban compared to rural areas. Whether this trend is driven by demand and the resultant increase in price or whether the overhead is driving overall cost in this context is unclear. However, the literature does not report this dynamic.

For UAE, there were only seven procedures over four years for the entire country, with no procedures at all in the midwest and west regions. This may indicate a glaring disparity in care access, availability (location) or utilization (preference/knowledge/beliefs/attitudes) when comparing urban and rural settings. This has also not been reported in the literature. However, the data do not indicate which proportion of the procedures in urban areas was undergone by rural residents. In addition to this, for UAE, the distribution of patients by race and region indicated that all of the seven rural UAE cases were white women, thus all the other groups were only urban. This indicates an absolute disparity in UAE for rural settings. Therefore, beyond conjecture it is unknown where and how rural women obtain UAE procedures, if and when they do. What drives this pattern is currently unknown.

A diligent search of the literature did not provide for comparison data on the prevalence of uterine fibroids by rural or urban distribution for this period. This agrees with conclusions by others that epidemiological data on fibroids were limited with very little reliable population-based data or research [55,56,57,58]. Thus, it is difficult to estimate the actual demand and supply gaps for rural residents during this period. Even though the literature points at a relatively similar overall cost after a year or more [31,48,40,30,59,23,38], it is unknown if the cost of UAE is driven by the rates of re-intervention. For this data set, hospital stays for the procedure indicate mostly a one day stay, including the day of procedure, and does not include follow-up.

The cost of hysterectomy was generally lower in the northeast and significantly higher in the urban than the rural settings, even though there were only three UAE procedures in the rural setting, all for White patients. The driving factors for these cost and service distributions are unclear.

The data do not contain points on some specific cost drivers, including the base demand for services and follow-up costs (for various reasons, including re-intervention). For this, the prevalence and incidence of uterine fibroids that will require either procedure are needed, as well as data indicating the residence of cases in the six months preceding the procedures.

### 5.2. Care Distribution

Results from the data indicate urban procedures were on average about nine times the number of rural procedures across the regions. Our data indicated a wide difference (about 59.83 to 1 untrimmed) in frequencies between hysterectomies and UAEs. Narayanan et al., 2016 [60], analyzing the Nationwide Inpatient Sample (NIS) data from 2012–2013, reported a ratio of 65 to 1, indicating a worsening trend from the 2004–2008 data. Also in our data, all UAE cases in the rural setting were White. In addition, a significantly larger number of hysterectomies were performed in the southern region. Several questions arise from this distribution: (1) What proportion of the urban procedures was from rural residents? (2) What effect will this have on care utilization; especially since the lack of resources may have an impact on the care-seeking behavior of rural women? (3) Because of the lack of data on the rural prevalence of uterine fibroids, what is the distribution of symptom severity and thus the need for more robust resource allocation/establishment in the rural setting? (4) How remote is this important gynecological care from rural women, and what are the drivers of this remoteness? (5) What is the state of awareness and experience in gynecological practice in the rural setting, what is the cost of this status, and what is needed to situate the benefits of improving gynecological care in the rural setting?

### 5.3. Racial Distribution

The glaring differences by race in the distribution of hysterectomies in the south call attention to determining the factors for such a very high proportion (92.08%) of hysterectomies performed on Black women in the rural south. Even though the data indicate that these women were referred by routine care, it is unclear if they underwent emergency procedures, indicating high severity. This may also point at the need to examine the indications for hysterectomy in the rural setting for Black women reporting (if they do) with fibroid symptoms to their care providers. There is the need to conduct appropriate research to determine the factors that drive this epidemiological profile in order to appropriately address and eliminate disparities.

#### 5.3.1. Primary Payer

The preponderance of primary payer for both UAE and hysterectomy were private/HMO or self-pay, while Medicare/Medicaid (public finance) came a distant second. This pattern points at a demographic that has adequate income to pay the premiums necessary to afford care. However, while not the purview of this study, it is unclear whether those who could not afford it also had access to Medicare and/or Medicaid, or whether they were utilizing needed care at all. This may be due to many factors, including knowledge of available services, access and material hardship.

#### 5.3.2. Household Income

By household income, especially in the rural setting, women in poorer households were more likely to have undergone hysterectomy procedures, with a predilection for the southern region. This is also intriguing in that most of the procedures are performed for women in households with low income in the rural south, with explanatory factors not being available in the data. It is also possible that rural women would avoid accessing care when they have fibroids, being afraid that their uteri would be removed, especially if they want more children. This may lead to underreporting of fibroids in those settings. In addition, nearly half of women in the rural setting who were treated with abdominal hysterectomy had household incomes below $38,999, compared to only 22.3% of urban women. The rate of hysterectomy in this group in the urban setting is comparable to a similar income group (23.57%) of urban women who had UAE. This may indicate that women who had higher household incomes tended to have UAE in the urban environment, while low income women in rural settings tended to have hysterectomies. This configuration is inexplicable, since, according to the U.S. Census Bureau [61,62], median urban household income was not significantly higher in the urban setting than the rural ($54,296 urban versus $52,386 rural), while there was significantly higher poverty rate in the urban than the rural setting (16.0% urban versus 13.3% rural overall, with 17.2% urban and 14.5% rural for women). A diligent search of the literature produced no reports relating income to hysterectomy and UAE procedures in this way. The rates observed may indicate that women tend to have much higher rates (49.92%) of abdominal hysterectomy in the rural areas, because they actually had far less choice, especially since very few UAE procedures were performed in the rural environment overall and there were no urban comparisons for rural UAE procedures. The popularity seemed to be apparent for total hysterectomy compared to that for UAE (131,865 vs. 1948). It is safe to assume that not all hospitals could offer the service of UAE, but nearly all hospitals could offer abdominal hysterectomy, contributing to cost difference. This may indirectly hint that only a few hospitals could provide the service of UAE. Thus, if the reason is not correlated to technology and/or facility, total hysterectomy might appear to be a better choice for symptomatic uterine fibroids.

Hospital discharge data were analyzed. Thus, the sample of women represents only those who engaged in care-seeking and does not include those who, for various reasons, did not. The findings can thus not represent all women, especially in the rural setting, since data, by variable or in frequency, are skewed towards the urban setting. Even though trimming outliers by cost and length of stay resulted in a smaller sample size, there was nevertheless an adequate residual sample representing 96.4% of the total number of both hysterectomy and UAE cases.

Descriptive analysis would not provide any inferential information; however, the purpose of this paper is descriptive, and thus no hypotheses are developed. Further questions raised by the descriptive paper will need to be addressed in the future.

The use of epidemiologic data that were narrow in scope and outdated—especially with regard to distribution of the incidence and prevalence of fibroids—without rural and urban differentiation, leaves critical gaps in cost and care settings. Critical gaps in current research and practice include up to date epidemiologic data on fibroids, rural-urban distribution of fibroids and distribution of gynecological services related to fibroids in the rural setting. This lack of equivalent prevalence of fibroids and the dearth of data on practice quality—especially for rural settings—may be masking significant issues specific to rural care access, quality, cost, utilization, efficiency, efficacy and effectiveness. Thus, updating research and surveillance information will help guide appropriate planning for interventional resources distribution and may help control costs.

## 6. Conclusions

Data from this study indicate that the preponderance of distribution of hysterectomies was skewed to the south, among poorer households, with rural Black women having a significant majority of their fibroids managed with hysterectomies. Despite the evidence in practice, the preponderance of research has been focused on the quality and cost of service, mostly based on non-current epidemiologic information sources [63,64,65,66], with a dearth of attention to care distribution. This is especially so with regard to care for women living in rural settings, where hardship of access may be an important consideration in many contexts, including that of cost. However, the factors and needs generating this picture are currently unclear.

Overall, it is important to note that the focus of research on hysterectomy and UAE for fibroids, and thus the purview of care, has been mostly on care quality efficiency and effectiveness. As a result, efforts and resources have been focused in this direction. Additionally, data related to disparities by location and availability have been missing in both the literature and the surveillance systems that capture the data. Also, the basic business wisdom of supply and demand has not necessarily been the focus of decision-making for care availability, considering that fibroids are already generally hyper endemic. The reasons for these are unclear, as the data analyzed showed significant gaps in variables captured to answer such questions. The data examined in this study indicated a significant disparity between rural and urban residents in both data collection and number of procedures conducted. Thus, the actual prevalence of uterine fibroids in the rural setting is unknown. Therefore, the rural burden of fibroids and fibroid care is not known, and neither is how this disparity affects utilization, accessibility, and self-efficacy for utilization among rural women. The large differences in racial patterns of patients undergoing hysterectomy for fibroids in the rural south need to be examined further. The causal factors and their dynamics have not been reported in the literature and are not within the purview of this paper. However, these are important considerations in cost control and effectiveness. Further research is needed to determine the background to differentials in cost and distribution of care among races, incomes, and between rural and urban settings. In addition, the absolute dearth in rural UAE procedures and the relative dearth of rural hysterectomies will require further assessment in surveillance and research, to determine the causality and provide redress where necessary for health care planning and cost control, and to eliminate disparities.

## Figures and Tables

**Table 1:** Regional Distribution of Uterine Artery Embolization (UAE) by Cost, Length of Stay, Race/Ethnicity, Adverse Complications, Primary Payer, Household Income and Patient Referral Source in the United States between 2004 and 2008.

	Procedure: Uterine Artery Embolization - UAE (*n* = 1948) *										
		Region									
		Northeast		Midwest		South		West		Overall (Total)	
		Urban	Rural	Urban	Rural	Urban	Rural	Urban	Rural	Urban	Rural
Sample (%) (N)		995	3	392	-	334	2	222	-	1943	5
Mean Cost ($)		19,979.66	30,307.88	20,431.99	0	20,985.01	13,169.94	26,533.59	0	20,992.56	23,452.70
Race/Ethnicity		995	3	392	-	334	2	222	-	1943	5
White		362	2	52	0	97	1	98	0	609	3
African American		366	0	91	0	156	0	40	0	653	0
Hispanic		69	1	0	0	23	0	20	0	112	2
Other		198	0	249	0	58	1	64	0	569	1
Primary Payer (N)		995	3	392	-	334	2	222	-	1943	5
Medicaid/Medicare/No Charge		94	0	57	0	31	0	26	0	208	0
Private/HMO, Self-Pay		894	3	329	0	282	2	186	0	1691	5
Other		7	0	6	0	21	0	10	0	44	0
Household Income (N)		995	3	392	-	334	2	222	-	1943	5
<$38,999		206	0	123	0	78	0	21	0	428	0
$39,000 to $47,999		133	0	91	0	92	0	48	0	364	0
$48,000 to $62,999		234	3	101	0	77	0	52	0	464	3
$63,000 or more		414	0	75	0	78	0	94	0	661	0
Missing		8	0	2	0	9	0	7	0	26	2

HMO: Health Maintenance Organization. * Numbers are after trimming outthe cost and length of stay outliers.

**Table 2:** Regional Distribution of Abdominal Hysterectomy (AHYS) by Cost, Length of Stay, Race/Ethnicity, Adverse Complications, Primary Payer, Household Income and Patient Referral Source in the United States between 2004 and 2008.

	Procedure: Abdominal Hysterectomy ( *n* = 131,865) *									
	Region									
	Northeast		Midwest		South		West		Overall (Total)	
	Urban	Rural	Urban	Rural	Urban	Rural	Urban	Rural	Urban	Rural
Sample (%) (N)	22,667	1277	23,497	3625	48,870	6103	23,048	1751	11,8082	12,756
**Mean Cost ($)**	**16,491.46**	**10,417.32**	**16,006.86**	**12,352.38**	**16,711.16**	**13,671.68**	**21,970.49**	**16,773.50**	**17,555.39**	**13,838.59**
**Race/Ethnicity (N)**	**22,667**	**1277**	**23,497**	**3625**	**48,870**	**6103**	**23,048**	**1751**	**118,082**	**12,756**
White	11,686	1158	6685	1870	17,127	2010	9602	959	45,100	5997
African American	5606	37	2490	93	14,949	1698	1673	16	24,718	1844
Hispanic	2507	43	129	42	5145	156	4048	145	11,829	386
Other	2868	39	14,193	1620	11,649	2239	7725	631	36,435	4529
**Primary Payer (N)**	**22,665**	**1277**	**23,494**	**3625**	**48,869**	**6103**	**23,048**	**1751**	**118,076**	**12,756**
Medicaid/Medicare/No Charge	3136	180	2404	414	4788	1155	2436	270	12,764	2019
Private/HMO, Self-Pay	19,199	1034	20,442	3082	41,158	4564	19,582	1370	100,381	10,050
Other	330	63	648	129	2923	384	1370	111	4931	687
**Household Income (N)**	**22,118**	**1251**	**23,347**	**3587**	**47,843**	**5854**	**22,520**	**1665**	**115,828**	**12,357**
<$38,999	4525	370	4514	1068	13,645	4120	3148	610	25,832	6168
$39,000 to $47,999	4283	539	5770	1853	12,058	1341	4851	727	26,962	4460
$48,000 to $62,999	5182	256	7040	625	11,988	326	6552	273	30,762	1480
$63,000 or more	8128	86	6023	41	10,152	67	7969	55	32,272	249
Missing	549	26	150	38	1027	249	528	86	32,271	399

* Numbers are after trimming out the cost and length of stay outliers.

**Table 3:** Mean Cost of Care for UAE and AHYS in the U.S.A. by Region and Location for the years 2004-2008 – Trimmed Data.

	Region	1 (Northeast)			2 (Midwest)			3 (South)			4 (West)			Tot		
	location	urban		rural	urban	rural		urban		rural	urban		rural	urban		rural
UAE	Mean (n)	$19,971.66 (995)		$30,307.88 (3)	$20,431.99 (392)	$0.00 (0)		$20,985.01 (334)		$13,169.94 (2)	$26,533.59 (222)		$0.00 (0)	$20,992.56 (1943)		$23,452.70 (5)
	diff, t (*p2*)	−$10,336.22 −$1.963 (0.050)			-			$7815.07 9.528 (0.002)			-			−$2460.14 −$0.575 (0.565)		
HYS	Mean (n)	$16,491.46 (22,667)		$10,417.32 (1227)	$16,006.86 (23,497)	$12,325.38 (3625)		$16,711.16 (48,870)		$13,671.69 (6103)	$21,970.49 (23,048)		$16,733.50 (1751)	$17,555.39 (118,082)		$13,383.59 (12,756)
	diff, t (*p2*)	$6074.14 *25.746 (0.000)			$3681.48 *30.408 (0.000)			$3309.47 *28.268 (0.000)			$5236.99 *21.212 (0.000)			$4171.80 *67.225 (0.000)		
UAE - HYS	Mean diff	$3480.20	$19,890.56		$4425.13		-	$4273.85	−$501.75		$4563.10	-		$3437.17	$10,069.11	
Both	Mean (n)	$16,638.14 (23,662)	$10,463.94 (1280)		$16,079.47 (23,889)		$12,325.38 (3625)	$16,740.17 (49,204)	$13,671.52 (6105)		$22,014 (23,270)	$16,733.50 (1,751)		$17,611.03 (120,025)	$13,387.54 (12,761)	
	diff, t (*p2*)	$6174.20 *26.011 (0.000)			$3754.09 *30.797 (0.000)			$3068.65 *28.485 (0.000)			$4223.49 *21.368 (0.000)			$4223.52 *53.433 (0.000)		

* Equal Variances Assumed; diff.: mean cost difference.
